# Representation of visual numerosity information during working memory in humans: An fMRI decoding study

**DOI:** 10.1002/hbm.25402

**Published:** 2021-03-11

**Authors:** Ian Morgan Leo Pennock, Timo Torsten Schmidt, Dilara Zorbek, Felix Blankenburg

**Affiliations:** ^1^ Neurocomputation and Neuroimaging Unit (NNU), Department of Education and Psychology Freie Universität Berlin Berlin Germany; ^2^ Center for Mind/Brain Sciences – CIMeC University of Trento Rovereto Italy; ^3^ Institute of Cognitive Science Universität Osnabrück Osnabrück Germany

**Keywords:** decoding, fMRI, MVPA, numerosity, short‐term memory, vision, working memory

## Abstract

Both animal and human studies on numerosity have shown the importance of the parietal cortex for numerosity processing. However, most studies have focused on the perceptual processing of numerosity. Still, it is unclear how and where numerosity information is coded when this information is retained during a working memory delay phase. Such temporal storage could be realized by the same structures as perceptual processes, or be transformed to a more abstract representation, potentially involving prefrontal regions. FMRI decoding studies allow the identification of brain areas that exhibit multi‐voxel activation patterns specific to the content of working memory. Here, we used an assumption‐free searchlight‐decoding approach to test where numerosity‐specific codes can be found during a 12 s retention period. Participants (*n* = 24) performed a retro‐cue delayed match‐to‐sample task, in which numerosity information was presented as visual dot arrays. We found mnemonic numerosity‐specific activation in the right lateral portion of the intraparietal sulcus; an area well‐known for perceptual processing of numerosity. The applied retro‐cue design dissociated working memory delay activity from perceptual processes and showed that the intraparietal sulcus also maintained working memory representation independent of perception.

## Graphical abstract

During an fMRI experiment, participants performed a retro‐cue delayed match‐to‐sample task in which the numerosity of visually presented dot stimuli were maintained in working memory. We found mnemonic numerosity‐specific activation in the right lateral portion of the intraparietal sulcus, which suggests that the IPS retained working memory representation independent of perception.

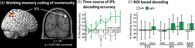

## 1 INTRODUCTION

Humans constantly estimate numerical quantities to inform decisions and to guide behavior: We walk into a room and estimate the number of people, or we estimate the number of coffee cups we would need for everyone in the lab. In the cognitive psychology literature, the number of elements, items, or separate objects a stimulus has, is referred to as “numerosity” (the equivalent to the mathematical term “cardinality” [Nieder, 2016]). The study of numerosity estimation is of particular interest because numerosity is an abstract concept and it is grounded in but not bound by sensory properties. For example, despite little or no perceptual commonalities, seven sounds, seven sticks, and seven steps all share the same semantic meaning “seven” (Eger, 2016; Nieder, 2016). However, unlike other abstract constructs (i.e., freedom) nonsymbolic numbers share characteristics with other sensory perceptions, that is, they are subject to adaptation, just like percepts of size, color, or speed (Burr & Ross, 2008). The study of numerosity processing has attracted major interest in animal and human studies, as it is considered a pre‐language ability, however, unbound from mere perceptual feature processing (Borghesani et al., 2018; Cavdaroglu & Knops, 2018; Lasne, Piazza, Dehaene, Kleinschmidt, & Eger, 2018; Nieder, 2013; Nieder, Diester, & Tudusciuc, 2006; Nieder, Freedman, & Miller, 2002; Nieder & Merten, 2007; Nieder & Miller, 2003; Nieder & Miller, 2004; Piazza, Izard, Pinel, Le Bihan, & Dehaene, 2004; Piazza, Pinel, Le Bihan, & Dehaene, 2007). It is thought that the ability to process numerosity information could be the foundation of performing complex mathematical operations (Feigenson, Dehaene, & Spelke, 2004; Gebuis, Cohen Kadosh, & Gevers, 2016), which requires numerosity information to be used by higher‐order cognitive processes. However, most previous works were focused on the perceptual aspects of the numerosity approximation process. In contrast, working memory (WM) studies, allow to test how information is temporally retained and thus held available for higher cognitive processes.

Previous research led to the description of the brain's approximate number system (ANS), as a set of brain regions involved in the estimation of numerosity (Feigenson et al., 2004; Gebuis et al., 2016; Nieder, 2016). To ensure that participants draw on the approximation of numerosity, as opposed to counting or other strategies, some conceptual distinctions are made. First, humans can process numerosity in two different formats: symbolic (e.g., Arabic numbers) and nonsymbolic, the latter requires an estimation of the numerosity through the ANS (Gebuis & Reynvoet, 2012; Pekár & Kinder, 2019). Furthermore, in order to investigate number estimation, it has to be ensured that participants do not use subitizing, which is the almost immediate, precise, and effortless (i.e., without counting) recognition of set sizes of up to 4–5 items (Cohen & Henik, 2016; Kaufman, Lord, Reese, & Volkmann, 1949; Trick & Pylyshyn, 1994). To investigate processes of the ANS, stimuli with numerosity above the corresponding subitizing threshold need to be used. Thirdly, stimuli need to be presented with an adequate time limit, as otherwise a task can be solved by counting the number of items.

Studies in humans and animals have revealed the intraparietal sulcus (IPS), prefrontal (PFC), and sensory cortices as main components of the ANS (Borghesani et al., 2018; Cavdaroglu & Knops, 2018; Harvey, Klein, Petridou, & Dumoulin, 2013; Lasne et al., 2018; Nieder, 2013; Nieder et al., 2006, 2002; Nieder & Merten, 2007; Nieder & Miller, 2004, Nieder & Miller, 2003; Piazza et al., 2007; Piazza et al., 2004). Most previous studies focused on the perceptual processing of numerosity, in which the importance of the parietal cortex for number processing has been demonstrated (e.g., Borghesani et al., 2018; Eger et al., 2009; Lasne et al., 2018). It was suggested that the IPS encodes the abstract concept of numbers because the activation of the IPS showed adaptation to numerical qualities in various types of number representation such as Arabic digits and dot stimuli (Piazza et al., 2007). The IPS has further been shown to activate when numbers are approximated (Feigenson et al., 2004) and was found activated in different number‐processing tasks such as number manipulation (Dehaene, Piazza, Pinel, & Cohen, 2003), presentation of numbers and letters in both auditory and visual modality (Eger, Sterzer, Russ, Giraud, & Kleinschmidt, 2003) and the IPS responds selectively when sets of items changed numerosity (Piazza et al., 2004). Finally, multivariate pattern analysis (MVPA) studies showed the importance of the IPS for number processing in a number judgment task (Eger et al., 2009; Lasne et al., 2018).

In addition to the IPS, nonhuman primate studies found number and numerosity codes in the lateral PFC (Nieder et al., 2002; Nieder & Miller, 2003; Nieder & Miller, 2004). Integrating reports on the parietal cortex and PFC, Bueti and Walsh (2009) proposed that these regions jointly realize a magnitude processing system for different quantities such as length, size, space, and time.

In addition to the IPS and PFC, several studies revealed numerosity information to be present in early sensory regions (Borghesani et al., 2018; Cavdaroglu & Knops, 2018; Lasne et al., 2018). As the numerosity of a stimulus is often linked to physical stimulus properties, it is likely that such codes reflect differences in low‐level stimulus properties (Gebuis et al., 2016). The difficulty is to dissociate low‐level stimulus features from a stimulus' numerosity, which requires careful stimulus and trial design (Gebuis & Reynvoet, 2011; Pekár & Kinder, 2019; Piazza et al., 2004; Salti, Katzin, Katzin, Leibovich, & Henik, 2017).

Taken together, the IPS and PFC are considered core regions of the ANS to generate an abstract representation of numerosity information, but the role of sensory cortices for numerosity processing might probably be limited to low‐level perceptual stimulus processing.

Most numerosity studies apply variants of comparison tasks, in which one stimulus is compared to a second stimulus presented shortly after each other. Those paradigms have similarities with delayed match‐to‐sample (DMTS) tasks as they are applied in the study of WM. DMTS tasks allow to study perceptual processing of numerosity and the potential conversion of sensory stimulus features to more abstract WM representations and have also been applied in previous human functional magnetic resonance imaging (fMRI) research (Lyons, Ansari, & Beilock, 2015; Lyons & Beilock, 2018). While DMTS tasks in electrophysiologic studies allow an assessment of the dynamics of stimulus processing, the slow evolution of the BOLD response in fMRI makes it challenging to dissociate if the activity is due to stimulus‐driven perceptual processes, from the activity that relates to the mental representation of a WM content; that is, the retention of numerosity information. To show that recorded BOLD activity in a DMTS task indeed relates to the WM representation, fMRI WM paradigms moved to implement corresponding experimental controls. Firstly, elongated WM delay periods are applied, to ensure that it is not stimulus‐driven BOLD activity that drives the main effect, but activity towards the later phase of a delay period; for example, delay periods of about 12 s are used. Secondly, a retro‐cue paradigm is applied in which two sample stimuli are presented, in which only one is memorized, and the second can be used in a control analysis to test for stimulus‐driven perceptual activity. Finally, introducing masking stimuli to overwrite perceptual residues, for example, suppressing afterimages, help to dissociate perceptual from WM‐related BOLD activation (Christophel, Hebart, & Haynes, 2012; Schmidt, Wu, & Blankenburg, 2017; Uluç, Schmidt, Wu, & Blankenburg, 2018; Wu et al., 2018).

Here, we used a WM DMTS paradigm in which the numerosity of visually presented dot stimuli had to be memorized to investigate which brain regions retain WM representations of numerosity information. The experimental design assured that participants retained an approximation of a stimulus' numerosity and not the stimulus layout. We used an assumption‐free searchlight decoding approach to test which brain regions exhibit numerosity‐specific activation patterns during the WM retention period. This approach allowed us to test in an unbiased way if IPS, PFC, or early visual cortex (EVC) are containing numerosity information during WM.

## 2 MATERIALS AND METHODS

### 2.1 Participants

Twenty‐four participants (14 males, 10 females; age: mean = 26.54 years, *SD* = 5.54) participated in the study but one participant was excluded due to excessive head motion (>15 mm) and therefore the data of 23 participants were included in the analysis. The participants did not report any neurologically or psychiatric disorder. All participants were right‐handed, assessed with the Edinburgh Handedness Inventory (Oldfield, 1971) with a mean laterality index of 79.64 (*SD* = 20.04). The local ethics committee of the Freie Universität Berlin approved the experimental procedure and each participant gave written informed consent and received monetary compensation or student points.

### 2.2 Experimental procedure

First participants were trained on the experimental task outside of the scanner. Then they performed the retro‐cue DMTS WM task in four runs of 18 min each, during fMRI scanning. After leaving the scanner they conducted a number naming test.

### 2.3 Stimuli

**FIGURE 1:**
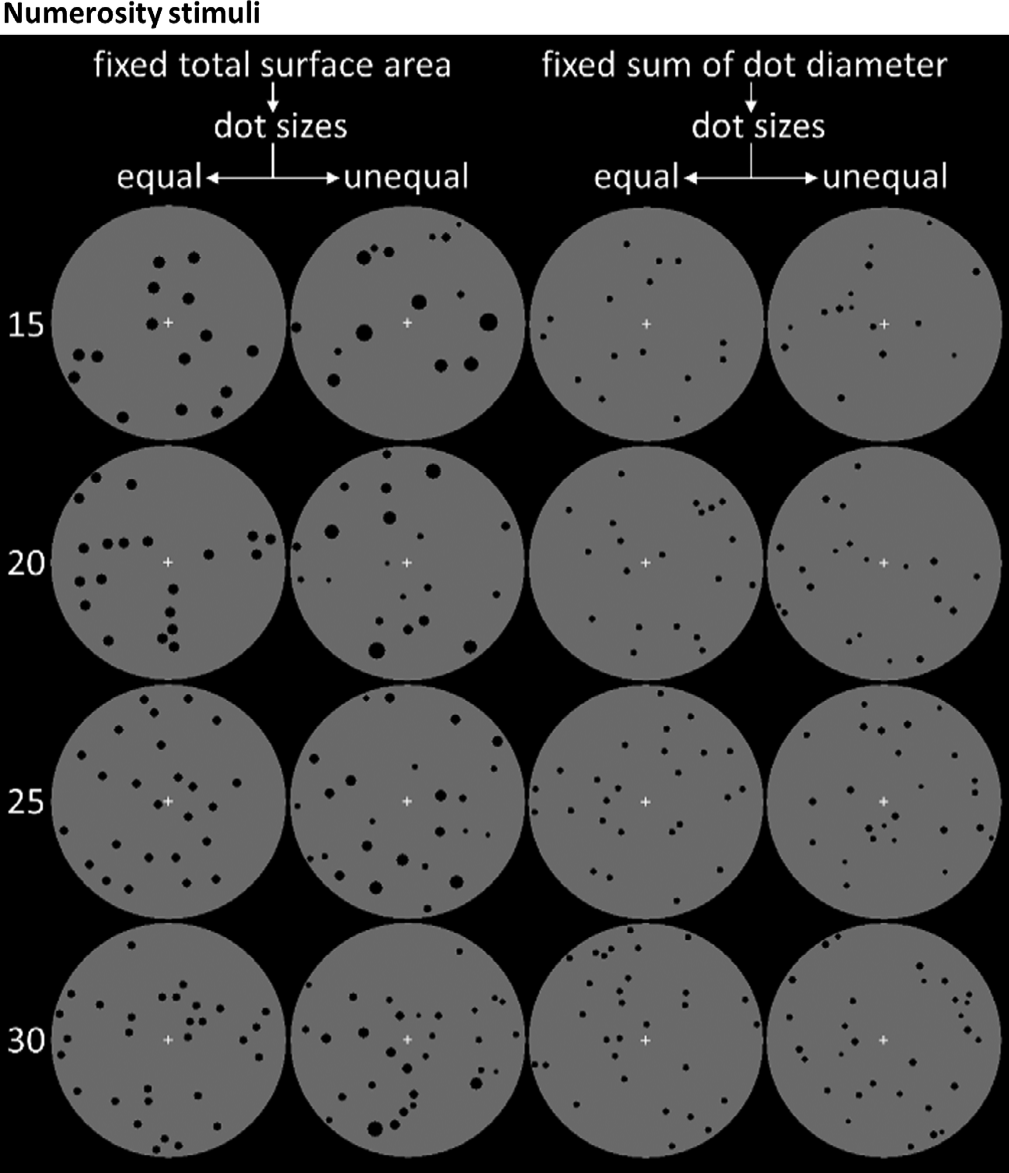
Numerosity stimuli. The to‐be‐remembered numerosities were presented as visual stimuli with different numbers of black dots on a gray circle. Four different numerosities were used for the to‐be‐memorized stimuli: 15, 20, 25, or 30. Participants did not know that only four different numerosities were used. Each applied stimulus had a different appearance. To exclude confounds of physical stimulus properties, for example, differences in total surface area or dot diameter, four different types of stimuli were used. Here, 16 example stimuli are displayed to illustrate the different numerosities and the various types of stimuli. The rows indicate the numerosities and on the columns the four categories are displayed: (a) fixed total surface area, equal dot size*s*; (b) fixed total surface area, varied dot size*s*; (c) fixed mean diameter, equal dot size*s*; (d) fixed mean diameter, varied dot sizes. Note that in each trial, the type of stimulus category was chosen randomly, thus rendering remaining stimulus differences not informative and necessitating to remember the numerosity for successful task performance

The visual dot arrays were presented as black dots within a gray circle in the center of the screen (see Figure 1). The background of the screen was black. Each stimulus was designed to contain a specific number of dots, in which the to‐be‐remembered numerosities were limited to four different numerosities due to MVPA requirements, namely: 15, 20, 25, and 30 dots. Participants were not aware that only stimuli with four different numerosities had to be memorized (see below).

One challenge in the use of visually presented numerosity stimuli is that one cannot match all stimulus parameters across stimuli. This is the case because parameters such as the total surface area of dots and the dot diameter are geometrically related to one another (Gebuis et al., 2016). We controlled for potential confounding effects of total surface area and dot diameter, by keeping either of them constant in half of the stimuli (a similar approach was introduced by Piazza et al. in 2004). Additionally, within these two categories, in half of the stimuli, the dots had equal sizes within a stimulus, and in the other half, the dot size varied within a stimulus but the total sum of their category (a total surface area or dot diameter) was kept constant. Four categories of stimuli resulted from these two variations: (1) fixed total surface area, equal dot sizes; (2) fixed total surface area, varied dot sizes; (3) fixed mean diameter, equal dot sizes; (4) fixed mean diameter and varied dot sizes (see Figure 1 for an example). Thereby, it was ensured that participants had to memorize the numerosity of a stimulus to perform the task, as memorizing any other stimulus property would not allow successful task performance. The use of these different stimuli renders any strategy that could be deployed besides estimating the numerosity very unlikely.

The position of dots was random. The dots did not overlap nor made contact. Three percent of the diameter of the stimuli (gray circle) was taken as a reference dot diameter to create the stimuli with fixed dot size. As a reference for the constant total surface area, we used the total surface area of the reference dot diameter multiplied by the largest numerosity used in the DMTS task. Note, different from Piazza et al. (2004), we matched the total surface area and dot diameter parameters based on the largest numerosity instead of using a single random value, to simplify the creation of the stimuli.

For the fMRI experiment, the stimuli were presented via a projector on a screen and participants saw the screen via a mirror system attached to the head coil. The resolution of the projection was set to 1,280 × 1,024 and the physical screen size to 33 cm by 24.7 cm, in 110 cm distance from the participants' eyes. The size of the gray circle was 4° in visual angle (diameter). For the training session outside of the MRI and the number naming test after the MRI session, stimuli were presented on a standard computer screen (size 37.8 × 30.1 cm) with the distance adjusted to present the stimuli in the same size of visual angle by ensuring the head position with a chinrest.

### 2.4 Experimental task

**FIGURE 2:**
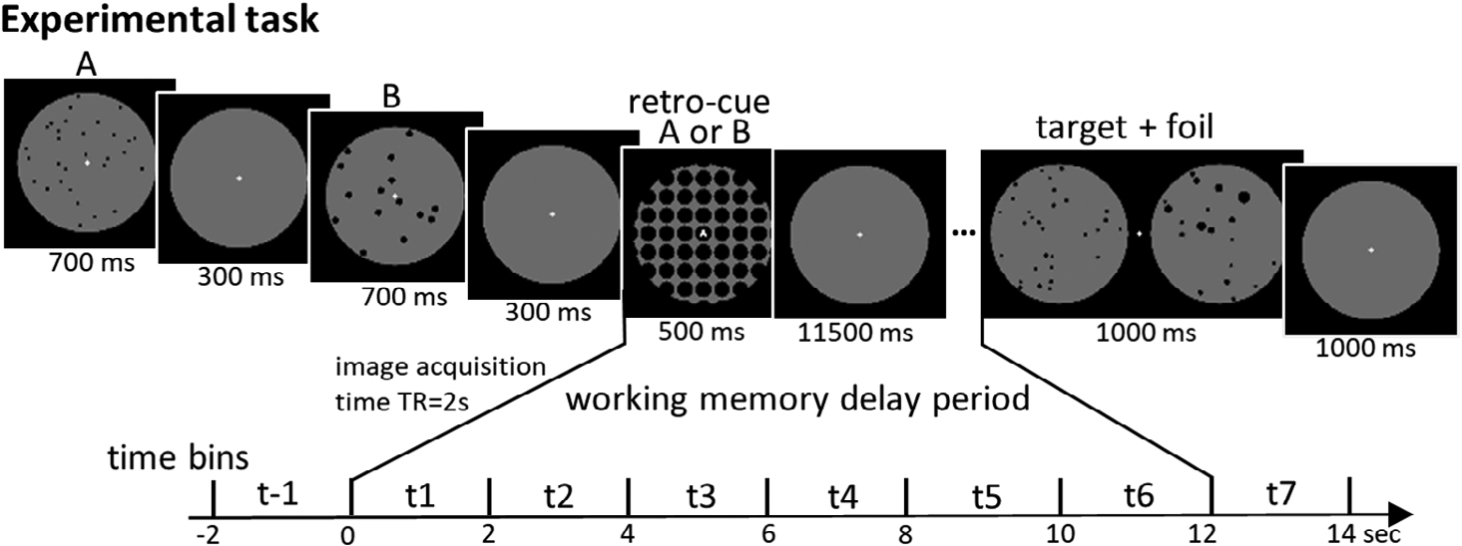
Experimental task. A DMTS WM task was applied in which two numerosity stimuli were displayed consecutively. A retro**‐**cue (the letter A or B in the center of the screen), presented together with a mask, indicated if the numerosity of the first or second stimulus had to be remembered. After the 12 s delay period a target and a foil stimulus were presented, and participants indicated with a left or right button press in which of these two stimuli the number of dots corresponded to the retained numerosity

The participants were subjected to a retro‐cue DMTS WM task (Figure 2), which was similar to the experimental paradigm employed in previous WM decoding studies (Christophel et al., 2012; Schmidt et al., 2017; Uluç et al., 2018; Velenosi, Wu, Schmidt, & Blankenburg, 2020; Wu et al., 2018). Two visual stimuli were presented consecutively, followed by a retro‐cue and a mask, indicating which numerosity had to be retained. The retro‐cue task allows to dissociate perceptual from retention processes. Whether the first or second stimulus had to be memorized was balanced within a run and each numerosity was memorized equally often as well as presented as a non‐memorized stimulus. After a delay period of 12 s the participants were presented with a target and a foil stimulus simultaneously, in which the center of the stimuli was 2.1° from the center of the screen. Participants had to indicate which of the two stimuli had the same numerosity as the one they memorized, by pressing the left or right button using their index or middle finger of their right hand. Targets were presented equally often on the left and right side for each participant, and the participants had 2 s to respond. The participants received feedback after every trial to keep up motivation in this demanding task. Furthermore, there is an increase in performance for numerosity discrimination when the ratio between numerosities is larger, which the Weber law accounts for (e.g., Izard & Dehaene, 2008). To make the given comparison task equally difficult for the four different numerosities, we used the Weber law to adjust the numerosity of the foil stimuli (Fechner, 1966), anchored at the mean of 22.5 + 12.375 dots per stimulus (Weber fraction: 12.375/22.5 = .55; same for all participants). This resulted in numerosity 15 having a lower foil stimulus of numerosity 10 and an upper foil of 23, the foils for numerosity 20 were 13 and 31; for numerosity 25 foils were 16 and 39 and for numerosity 30 foils were 19 and 47. For each run the number of applied stimuli of the four stimulus categories (see above) were balanced. Every participant had their own unique stimulus set, to prevent any possibility of a random bias and thus no stimulus was used twice within the study. In each trial, the displayed stimuli were chosen equally often from the four categories. Each run contained 48 trials, 12 for each of the four numerosity conditions, interleaved by a 2 or 4 s interstimulus interval. Additionally, 12 catch trials were included with a delay period of either 4 or 8 s to ensure an active WM representation was sustained throughout the delay period (Christophel et al., 2012; Schmidt et al., 2017).

Before the fMRI session, participants were trained outside the scanner and one full run was conducted. Participants were included in the study, when performing above 65% accuracy.

### 2.5 Number naming test

To test that the participants had not counted the number of all dots in the applied stimuli, we performed a number naming test after the participants left the MRI scanner. To assess the highest numerosity in our stimulus set for which participants were able to name the exact number of dots, we used a modified version of a number naming test applied in Spitzer, Fleck, and Blankenburg (2014) and Uluç, Velenosi, Schmidt, and Blankenburg (2020). In each trial participants were presented with one stimulus, for 700 ms as applied in the DMTS task, and had to report the exact number of dots by typing it in on a keyboard. The number naming test took approximately 20 min and comprised 288 trials: 24 trials each for numerosities close to the typical subitizing threshold, that is, {5, 6, 7, 8, 9, 10, 11} and 12 trials each for those numerosities in the higher and lower end, that is, {3, 4, 13, 15, 16, 20, 25, 31, 38, 47}. This range covered the numerosities presented in the DMTS task and allowed us to fit sigmoidal curves to each participant's performance data and consequently determine 50%‐correct naming thresholds. Note, this measure does not directly reflect the subitizing threshold, as that would require applying masking after stimulus presentation and the measuring of reaction times (Burr, Turi, & Anobile, 2010). The applied 50%‐naming threshold will usually be above the subitizing threshold.

### 2.6 fMRI data acquisition and preprocessing

Functional MRI data were acquired with a 3 T Siemens Tim Trio system and a 32‐channel head coil. Each participant did four runs of 18 min, comprising 540 functional images each (T2*‐weighted gradient‐echo EPI: 37 slices; ascending order; 20% gap; whole brain; TR = 2000 ms; TE = 30 ms; 3 × 3 × 3 mm^3^; flip angle = 70°; 64 × 64 matrix). After the four runs, structural MRI data were acquired (MPRAGE, 176 sagittal slices, TR = 1900 ms, TE = 2.52 ms, 1 × 1 × 1 mm^3^ voxel).

The preprocessing of fMRI data was performed in SPM12 (Wellcome Trust Centre for Neuroimaging, Institute of Neurology, University College London, London, UK). Preprocessing was limited to realignment to preserve the spatiotemporal structure of the fMRI signal as much as possible for the classification analysis. No corrections for field distortions were applied. We used finite impulse response (FIR) models to obtain run‐wise beta estimates for each WM numerosity condition time‐resolved over the delay period. The start of the trials and the acquisition of functional images was time‐locked. We, therefore, modeled the 12 s delay period as six consecutive time bins as FIR regressors. We included one‐time bin before and after the WM delay which totals to eight time bins modeled for each condition. Catch trials were not modeled as they had shorter WM delays and were only included to assure that participants keep their WM representation active as they could not know when exactly a target stimulus was presented. As catch trials were not informative for the main analysis and the order of trials was randomized, they did not systematically affect the regressors of interest. The first‐level model used high pass filtered data (cut‐off of 128 s) and included 132 regressors (4 conditions x 4 runs x 8 time bins + 4 run constants).

### 2.7 Multivariate searchlight decoding

To identify which brain areas exhibit numerosity‐specific activation patterns during the delay period, we used a time‐resolved multivariate searchlight decoding approach (Christophel et al., 2012; Kriegeskorte, Goebel, & Bandettini, 2006). All MVPA analyses were performed using the decoding toolbox (TDT; Hebart, Görgen, & Haynes, 2015), which used LIBSVM (Chang & Lin, 2011). A support vector machine (SVM) classification was used with a cross‐validation scheme for the four runs. We used the linear SVM classifiers to distinguish between two types of activation patterns, thus implementing a pairwise‐classification scheme. Independent whole‐brain searchlight analyses were performed for each of the six possible pairs of retained numerosities ([15,20], [15,25], [15,30], [20,25], [20,30], [25,30]). For each pairwise classification, beta estimates from a four‐voxel radius sphere were extracted for a pair of memorized sample stimuli and z‐scaled (normalized) across the samples for each voxel. Data of three runs were used to train a classifier and its generalization was then tested on the remaining run (leave‐one‐out cross‐validation). The center of the searchlight was moved voxel‐wise through the brain and thereby whole‐brain accuracy maps for each pair of beta maps were derived. These reflect how accurate the classifier can separate the two WM contents based on the given activation patterns. These six accuracy maps were averaged within time bins, normalized to MNI space using unified segmentation, and smoothed with an 8 mm full‐width half‐maximum kernel.

Mean accuracy maps were entered to a second level ANOVA (repeated‐measures across time bins) design, using the flexible factorial design specification of SPM12. We computed a t‐contrast to test decoding accuracies in each voxel against 50% chance level to determine if a voxel contained information on the stimulus identity across the delay phase. The chance level is 50% as the chance level of each of the pairwise‐classification steps is 50%. The t‐contrast was computed for time bins t2–t7 (corresponding to the 2–14 s of the delay phase, see Figure 2) to account for the delayed BOLD response and to model only WM time bins after the retro‐cue was presented (Christophel, Cichy, Hebart, & Haynes, 2015). Significant voxels were reported with a threshold of *p* < .05 family‐wise error corrected (FWE) for multiple comparisons at the voxel level. Reported coordinates correspond to MNI space.

### 2.8 Region‐of‐interest based decoding analysis

Following the a priori hypotheses that EVC, IPS, and PFC might code numerosity information during WM and in addition to the main searchlight‐analysis, we conducted a region‐of‐interest (ROI) analysis. We used 12 ROIs from the Anatomy Toolbox (Eickhoff et al., 2007), namely left and right ROIs of the hOc1, hOc2, hIP1, hIP2, hIP3, and BA44. For the ROI based decoding analysis, we used the beta estimates from the same FIR models as in the main analysis. Beta‐images were normalized to MNI space and MVPA was conducted with TDT in a time‐resolved fashion as in the main analysis.

### 2.9 Control analysis: Univariate parametric modulations

To test if in addition to the multivariate effects, also univariate differences between numerosity conditions could be detected, we tested for parametric modulation of BOLD activity by the retained numerosity. For each participant, new first‐level models with HRF convolved regressors were formulated. Firstly, the realigned data were normalized to MNI space and smoothed with an 8 mm full‐width half‐maximum kernel. Then we modeled the following regressors: four onset regressors modeling the to‐be‐remembered numerosity stimuli presentations, an onset regressor for the retro‐cue, four boxcar regressors for the 12 s WM delay period separately (one for each numerosity), one onset regressor modeling the presentation of the target + foil stimuli, and two onset‐regressors for left and right button responses. A parametric first‐level contrast (−1.5–0.5 0.5 1.5) was computed across the four numerosity WM regressors and corresponding contrast images forwarded to a second level one sample *t*‐test.

### 2.10 Control analysis: Decoding the non‐memorized stimuli

We conducted a control analysis for the specificity of the main analysis, testing for above‐chance decoding accuracy for the non‐memorized stimulus. New FIR‐models were estimated, with four sets of FIR regressors that modeled the trials in which a stimulus was presented but not memorized. Each beta image was estimated with an equal amount of data (the same number of trials) as in the original analysis. Beta‐images were entered in the exact same SVM searchlight and second‐level analysis as the main analysis.

## 3 RESULTS

### 3.1 Performance in DMTS task

**FIGURE 3:**
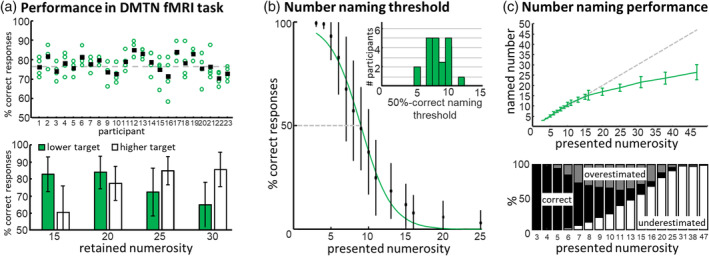
Behavioral assessment. (a) Participants performed consistently above‐chance across all four runs of the DMTS fMRI task. Open circles represent the performance in each of the four runs; filled squares represent the overall mean performance. When assessing the performance across the different numerosities and with regard to whether a lower or higher foil stimulus was presented along with the target stimulus (means ± *SD*), characteristic differences in performance levels can be seen which indicate a “regression to the mean” effect. Trials with higher foil stimuli show increasing performance with increasing numerosity, and the opposite effect is seen for trials with lower target stimuli. This effect is well known from working memory studies on abstract quantities. Over the delay period, the mental representation of a number appears to slightly drift towards the overall mean of the stimulus set thus the comparison of a stimulus of numerosity 15 (WM representation is biased to the mean, meaning higher than 15), with a lower foil stimulus is getting easier. On the other hand, a comparison to a higher foil tends to get more difficult. Overall, these effects are expected and do not confound the main fMRI analysis, as the WM representation of all stimuli is equally affected by the regression to the mean effect. (b) A number naming test was performed outside of the fMRI scanner to assess a threshold measure up to which the number of dots in a stimulus could be determined exactly. Group performance is displayed as percentage of correct number naming (means ± *SD*) and a sigmoidal curve fitted to the data. Data are only shown until the numerosity of 25; the full range was: [3–48]. The distribution of individual thresholds is displayed in the figure inset. The group average of the 50%‐correct naming threshold was between eight and nine. As the lowest to‐be‐remembered numerosity applied in the DMTS task was 15, these results confirm that participants were not able to count all the dots in a stimulus but had to rely on an estimation of the number of dots. (c) As reported in previous number naming tests, participants tend to underestimate the number of dots for higher numerosity (upper panel). The lower panel shows the distribution of correct naming (black), those trials in which the number of dots was overestimated (gray) and those underestimated (white)

All participants responded correctly in more than 70% of the WM trials with an average performance of 77.43 ± 4.14% (mean ± *SD*; see Figure 3a). The average performance in the catch trials was 80.30 ± 5.60%. Testing for potential performance differences across numerosity conditions and runs with a 4 × 4 repeated‐measures ANOVA revealed no significant differences between runs (*F* [3, 66] = 0.07, *p* = .97, *η̂* ^*2*^_*p*_ = 0.003), and no significant interaction (*F* [9,198] = 0.88, *p* = .54, *p* = .97, *η̂* ^*2*^_*p*_ = 0.04). A main effect of condition became significant (*F* [3, 66] = 9.97, *p* > .001, *p* = .97, *η̂* ^*2*^_*p*_ = 0.31) and post hoc analyses indicated the numerosity 15 (72.08 ± 9.02%) condition was more difficult than the numerosity 20 (81.88 ± 5.55%) and 25 condition (79.96 ± 6.24%) and the numerosity 30 condition (75.81± 6.17%) was more difficult than the numerosity 20 condition; Pairwise post hoc Tukey's HSD tests: 15 versus 20: *p* > .001; 15 versus 25: *p* > .001; 20 versus 30: *p* > .05. Trials without response (no response was given or responded too late) were excluded from the behavioral analysis, in which no participant missed more than 10% of WM trials and only one participant missed more than 6 of the 198 trials across the four runs.

We further investigated the significant main effect of the condition by including a possible effect of the upper or lower foil stimulus being shown in the trials. We conducted a 4 × 2 repeated‐measures ANOVA with factors numerosity condition and displayed foil stimulus and found a main effect of condition (*F* [3, 66] = 9.92, *p* > .001, *η̂* ^*2*^_*p*_ = 0.31) and a significant interaction between the condition and displayed foil stimulus (*F* [3,66] = 42.54, *p* > .001, *η̂* ^*2*^_*p*_ = 0.66). No main effect was found for displaying a lower or higher foil stimulus (*F* [1, 22] = 0.18, *p* = .68, *η̂* ^*2*^_*p*_ = 0.008). Post hoc analyses revealed that in the numerosity condition 15 (lower: 82.97 ± 10.12%; higher: 61.08 ± 15.47%) and 20 (lower: 85.23 ± 8.68%; higher: 78.47 ± 9.51%) the target was more easily detected when the foil stimulus was lower than when the foil stimulus was higher than the target. For the numerosity conditions 25 (lower 73.68 ± 14.69%; higher: 86.39 ± 8.06%) and 30 (lower: 65.52 ± 13.16%; higher: 86.15 ± 10.01%) the reverse was true; Pairwise post hoc Tukey's HSD tests: 15 upper versus lower: *p* > .001; 20 upper versus lower: *p* = .03; 25 upper versus lower: *p* > .005; 30 upper versus lower: *p* > .001.

### 3.2 Number naming test

We excluded the data of two participants from the analysis of the number naming test, as in that task they showed a strong bias to report the number of dots in a stimulus as a multiple of five (the data of these participants was kept for the fMRI analysis as the number naming task was performed after the fMRI task and this bias should be unrelated to the performance in the fMRI WM task). Across the remaining *n* = 21 participants, the 50%‐correct naming thresholds ranged from 6 to 13 (mean: 8.94 ± 1.71 *SD*; Figure 3b). As reported in previous studies (Crollen, Castronovo, & Seron, 2011), also in our sample the number of dots was underestimated for higher numerosities as displayed in Figure 3c.

### 3.3 MVPA

**FIGURE 4:**
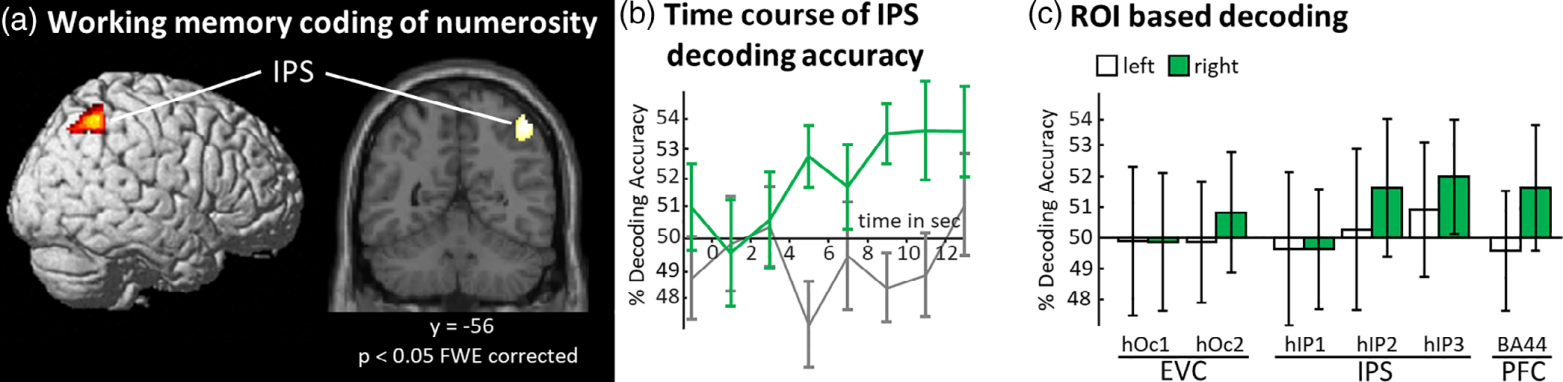
WM coding of numerosity. (a) Brain regions which showed activity patterns while numerosity is maintained in WM, revealed by an assumption‐free whole‐brain searchlight decoding approach. One cluster of above‐chance decoding accuracies in the right intraparietal sulcus (IPS; x = 46, y = −54, z = 54 mm, z‐score = 5.29, cluster size = 277) was revealed by a t‐contrast across the WM delay period, at *p* < .05 FWE corrected. This cluster was mainly assigned to the hIP3 subregion of the IPS based on probability maps in the Anatomy Toolbox (Eickhoff et al., 2007). No above‐chance decoding was found in early visual cortices (EVC) or in the prefrontal cortex (PFC) even when the threshold was lowered to *p* < .001 uncorrected. (b) The time course of the decoding accuracies through the delay period were extracted for the peak voxel of the IPS (mean ± *SEM*). The green line indicates decoding accuracy of the main analysis and the gray line displays the decoding accuracies for the control analysis. (c) Results of region of interest (ROI) based decoding analysis for regions with a priori hypotheses, namely EVC, IPS subregions and the PFC (mean ± *SEM*). Bars show the mean decoding accuracy for the time bins t2–t7, as used for the contrast of the main analysis displayed in (a). In line with the searchlight analysis, the right IPS displayed the strongest above‐chance decoding accuracies, wheras EVC did not show above‐chance decoding. Testing with one‐sample one‐sided *t*‐tests, only the right hIP3 of the ROI analyses exceeded significance (*p* < .05). Interestingly the right PFC (BA 44) also displayed a trend toward above‐chance decoding

MVPA was used to decode the content of WM in order to identify the brain areas in which numerosity‐information is retained during the WM delay. We computed a t‐contrast across six‐time bins of the WM delay period (mean of bins t2–t7; corresponding to the 2–14 s). With a threshold of *p* < .05 FWE corrected on the voxel level (cluster extent threshold of 10 voxels), we found one cluster of above‐chance decoding accuracies in the right parietal cortex, in particular the IPS (x = 46, y = −54, z = 54 mm, z‐score = 5.29, cluster size = 277; see Figure 4a). The cluster was assigned to the hIP3 with 24.7%, the PGa with 15.8%, PFm with 10.9%, and 7PC with 5.1% according to the Anatomy Toolbox (Eickhoff et al., 2007). No above‐chance decoding was found in EVC or PFC even at a more liberal threshold of *p* < .001 uncorrected. The temporal evolution of decoding accuracies across the WM delay period, for the peak voxel of the identified IPS cluster is presented in Figure 4b. This time course showed that decoding accuracy evolved from chance level with the approximate temporal profile of the hemodynamic response function to reach a maximum and level off. As decoding accuracy remained above‐chance level until the end of the delay period, this decoding accuracy was ascribed to the WM representation and not to perceptual processes, for which a return of decoding accuracies to chance level during the WM delay would have been characteristic.

### 3.4 Region‐of‐interest based decoding analysis

In addition to the assumption‐free searchlight‐analysis, we tested if information on retained numerosities can be decoded from a priori defined anatomical ROIs in EVC, IPS, and PFC. To check for above‐chance decoding, we tested mean decoding accuracies for the WM delay period (mean of bins t2–t7; corresponding to 2–14 s after the delay period onset) against zero with one‐sample one‐sided *t*‐tests: left hOc1: (M = −0.11, *SD* = 5.93% decoding accuracy above‐chance level of 50%), t(22) = −0.09, *p* = .53; right hOc1 (M = −0.14, *SD* = 5.43), t(22) = −0.12, *p* = .55; left hOc2 (M = −0.14, *SD* = 4.76), t(22) = −0.14, *p* = .55; right hOc2 (M = 0.82, *SD* = 4.78), t(22) = 0.82, *p* = 0.21; left hIP1 (M = −0.36, *SD* = 6.06), t(22) = −0.29, *p* = .61; right hIP1 (M = −0.36, *SD* = 4.76), t(22) = −0.36, *p* = .64; left hIP2 (M = 0.27, *SD* = 6.36), t(22) = 0.20, *p* = .42; right hIP2 (M = 1.63, *SD* = 5.44), t(22) = 1.44, *p* = .08; left hIP3 (M = 0.92, *SD* = 5.33), t(22) = 0.83, *p* = .21; right hIP3 (M = 1.98, *SD* = 4.53), t(22) = 2.10, *p* = 0.02; left BA 44 (M = −0.42, *SD* = 4.76), t(22) = −0.43, *p* = .66; right BA 44 (M = 1.62, *SD* = 4.96), t(22) = 1.56, *p* = .07 (see Figure 4c). The right hIP3 showed a significant difference from chance (*p* < .05) and the right hIP2 (*p* = .08) and BA44 (*p* = .07) showed trends toward above‐chance decoding.

### 3.5 Control analysis: Univariate parametric modulations

To test for any brain region to display increasing activity with increasing numerosity, we tested for a corresponding parametric modulation with a second‐level one sample *t*‐test. No significant voxels were revealed at *p* < .05, FWE corrected, nor at a more liberal threshold of *p* < .001 (cluster extent threshold of 10 voxels).

Not finding a parametric univariate effect suggests our MVPA analysis was not driven by a difference in average BOLD activation per condition.

### 3.6 Control analysis: Decoding the non‐memorized stimuli

As for the control analysis, namely decoding the non‐memorized stimulus, we did not find any significant activation clusters with the same thresholding as in the main analysis.

## 4 DISCUSSION

Here we applied a WM task in which the numerosity of visually presented stimuli had to be retained. We tested throughout the whole brain for regions that contain WM codes of numerosity using MVPA. We found numerosity‐specific activation patterns in the right parietal cortex, in particular in the human intraparietal area 3 (hIP3) of the IPS. Our control analysis demonstrated the specificity of the main finding, as decoding the non‐memorized numerosity did not reveal any significant clusters. Our study extended the investigation of numerosity processing to the domain of WM and revealed that the IPS is not only a core region for the perceptual processing of numerosity but also for the retention of numerosity information, on the contrary, we did not find sufficient evidence for numerosity codes in the PFC and no evidence for numerosity WM codes in EVC.

### 4.1 Behavioral assessment and experimental control

Task performance indicated that numerosity information was processed and successfully retained for the WM delay period. Participants performed consistently across the four experimental runs, demonstrating that the different numerosities were estimated and retained accurately throughout the experiment. Participants performed equally well in catch trials with shorter WM delays, indicating that the WM representation was held active throughout the delay period.

Our behavioral analysis revealed performance differences between the to‐be‐remembered numerosities. When investigating differences in the trials in which a higher or a lower foil stimulus was presented, the behavioral data showed evidence for a “regression to the mean” effect (also called “time order effect”; Ashourian & Loewenstein, 2011; Herding, Spitzer, & Blankenburg, 2016; Karim, Harris, Morley, & Breakspear, 2012; Preuschhof, Schubert, Villringer, & Heekeren, 2010). This is a common finding in WM studies in which quantities are retained (e.g., vibratory frequencies; compare Herding et al., 2016). This data provided additional evidence that participants did not remember a verbal label of a number or any other fixed numeric code, instead, they most likely remembered an approximation of a quantity which is subject to the regression to the mean effect. As trials with all different numerosities are equally affected by this, and as our main decoding analysis was performed for every time bin independently, the findings of our main analysis should not be affected.

After the fMRI scanning we applied a number naming test to confirm that the participants did not rely on subitizing and used the ANS to derive their WM representation. The number naming task ensured that the applied stimuli had numerosities for which participants were not able to name the exact number of dots in the stimuli. We used the 50%‐correct naming threshold as a pragmatic measure for countability. This measure did not directly reflect the subitizing threshold (Feigenson et al., 2004), however, it assured that participants were not able to count the dots in the stimuli. The threshold was found to be around nine dots and all participants' thresholds were well below 15, which was the number of dots in the stimulus with the lowest retained numerosity. Taken together this demonstrates that participants estimated the numerosity of stimuli and did not count nor used subitizing.

A challenge for the design of visual dot numerosity stimuli is that one cannot change the numerosity and hold all other stimulus' parameters constant. As a consequence, stimuli can contain sensory cues, which are physical stimulus properties that are nonnumerical, such as total surface area or dot diameter (Gebuis et al., 2016; Pekár & Kinder, 2019; Piazza et al., 2004; Salti et al., 2017). The dot position in our stimuli was chosen randomly, which does not exclude minor differences in the convex hull, namely the area within a stimulus covered with dots. However, our stimuli and experimental design ensured that total surface area and dot diameter size were not informative to perform the task. As each trial had four stimuli displayed that were randomly chosen from the four categories, there was no consistency in the display of these nonnumerical stimuli properties for each trial. By using four categories of stimuli (see Figure 1 and Methods), participants were most likely retaining an abstract numerosity representation, instead of memorizing any sensory stimulus features because the stimuli were randomized, and no particular strategy could be used besides estimating the numerosity. In addition, the application of a visual mask to overwrite any peripheral stimulus residues and the retro‐cue paradigm were designed to foster that an abstract numerosity estimate was remembered. If the IPS would represent perceptual features, this would show in the MVPA analysis of the non‐remembered stimuli. The reasons mentioned above leave us confident, that the careful task design and control analysis make sure that the main finding of our analyses reflect codes of abstract numerosity.

### 4.2 Working memory codes of numerosity

Our study revealed numerosity WM codes only in the IPS but not in the EVC or PFC. We did not find univariate activation differences between the four numerosity WM conditions, when testing for parametric modulations of numerosity throughout the brain. This indicates that it is not a mere activation increase for higher numerosities. Instead, the numerosity appeared to be represented by distributed neural populations, which contributed to a multi‐voxel activation pattern. The IPS has previously been identified as a core region within the ANS for numerosity and number processing (e.g., Borghesani et al., 2018; Eger et al., 2009; Lasne et al., 2018; Lyons et al., 2015; Lyons & Beilock, 2018; Piazza et al., 2007). This has been shown for paradigms in which numerosity information was presented in different modalities (Eger et al., 2003) and used within different types of tasks (Dehaene et al., 2003; Eger et al., 2003; Lyons et al., 2015; Piazza et al., 2004). In WM literature, it has been a central question whether and to what extend the same regions and neuronal codes can be found during perception as well as WM delay phases (Christophel, Klink, Spitzer, Roelfsema, & Haynes, 2017; Pasternak & Greenlee, 2005; Xu, 2017; Xu, 2018). While some mental material is retained in perceptual codes, other material seems to be transformed in other types of codes and stored in higher‐order cortices (Christophel et al., 2017). It is assumed that the level of abstractness a mental representation has, relates to the processing properties of a brain region and its corresponding level in the cortical hierarchy. One assumes that a mental representation which is retained in a sensory‐like format (low level of abstraction) can be decoded from EVC, whereas more abstract mental representations (e.g., in multimodal, categorical, conceptual, language‐like, or symbolic formats) should be decodable from higher‐order cortices which are known to process corresponding types of information (Christophel et al., 2017). Along this line of argumentation, finding WM codes in the IPS as a hierarchically higher‐order region rather than the EVC would suggest that the WM representation is stored in a “more abstract” type of code than sensory cortices would process. Taken together, the finding that the IPS exhibits numerosity WM codes is in line with the view that the IPS contains a modality independent code of numerosity. A WM representation thereof is maintained in a format suited to the task demands; in the given task this would not be sensory‐like codes and therefore not found in EVC but in the IPS.

The reported time course of the evolution of decoding accuracies over the WM delay period provided further evidence for the role of the IPS in WM, as after a build‐up of decoding accuracies, it remained above‐chance until the end of the retention period. If the IPS only represented perceptual processes, one would expect that decoding was possible only shortly after stimulus presentation and would thereafter return to chance level, which was not the case here.

Previous studies have demonstrated the role of the IPS to the perceptual processing of numerosity. In addition, the IPS is also well‐known to contribute to WM processes and yet there might be a functional distinction between the medial and lateral parts of the IPS. The medial bank of the IPS generally processes perceptual information and has a visual topographical organization (Mackey, Winawer, & Curtis, 2017; Swisher, Halko, Merabet, McMains, & Somers, 2007). Our findings in both, the searchlight and ROI analyses, showed WM activity patterns in more lateral parts of the IPS, which can also be seen in other visual WM studies (Bettencourt & Xu, 2016; Sheremata, Somers, & Shomstein, 2018; Somers & Sheremata, 2013; Wu et al., 2018; Xu, 2007). That our study found more lateral parts of the IPS might be because more medial regions prefer low numerosities and lateral areas prefer higher numerosities (Harvey et al., 2013). With the given limited spatial resolution of the given study, we can only speculate on this possible distinction as our study does not allow to make direct comparisons with perceptual processing of numerosity. Future high‐resolution fMRI studies could reveal if there are indeed different subdivisions of the IPS that contribute to perceptual processing or WM representations.

Our study did not reveal numerosity codes in sensory regions, namely EVC. No effects were found in the searchlight‐analysis nor the ROI approach. The role of sensory cortices for WM retention has been intensely discussed in recent WM literature (Xu, 2017; Xu, 2018). As no final consensus has been reached, there is some convergence onto the view that sensory cortices only rarely code WM‐related information (Christophel et al., 2017; Pasternak & Greenlee, 2005; Xu, 2017; Xu, 2018). As different regions appear to jointly code WM content, sensory cortices appear to be only relevant when sensory‐type information is retained, but not when more abstract types of information are retained (Christophel et al., 2017; Schmidt & Blankenburg, 2018; Schmidt & Blankenburg, 2019). A recent fMRI numerosity study found comparable decoding accuracies in EVC and the IPS during a delayed numerosity comparison task. However, only the IPS showed a correlation between fMRI decoding performance and the subjects' behavioral precision in a numerical discrimination task (Lasne et al., 2018). Another study conducted a DMTS task and displayed visual dot arrays with the numerosities 1 till 9. With a representational similarity analysis, the researchers showed that the neural patterns were also in the bilateral IPS (Lyons et al., 2015). Crucially, these studies were not specifically designed to reliably dissociate the perceptual processing from the numerosity representation during the delay period. Our retro‐cue paradigm with the applied control analyses dissociates the WM representation from the perceptual processes. Not finding sensory regions is therefore in line with the current WM literature, that higher‐order abstract stimulus attributes such as numerosity, are not represented in sensory regions.

Our study did not find numerosity codes in the PFC. The searchlight analysis did not reveal above‐chance decoding even at an uncorrected threshold of *p* < .001. The ROI analysis showed a trend in the right PFC namely in BA 44, which was however not significant. This null finding is in contrast with multiple nonhuman primate studies which repeatedly found PFC codes of numerosity including WM tasks (Jacob, Hähnke, & Nieder, 2018; Nieder et al., 2002; Nieder & Miller, 2004). For example, Nieder et al. (2002) recorded 352 PFC neurons and 111 neurons showed numerosity‐specific activation during a memory delay. With regard to our null finding in the PFC, it is well possible that also neurons in the human PFC show such activity patterns, as corresponding signals were not possible to be detected with the limited spatial resolution of the fMRI voxel level. It is comprehensible that neuronal populations in the PFC exhibit WM codes of numerosity, but these populations are not distributed with a suited sparsity to elucidate different voxel activation levels, which in turn would be detectable with MVPA (Haynes, 2009). Future research with high‐resolution fMRI might contribute further insights if WM numerosity codes in the PFC are indeed absent in humans.

Interestingly, multiple human studies found PFC codes when testing for numerosity‐like information of sequentially presented stimuli. In such stimuli, the to‐be‐remembered information was presented as series of pulses, for example, as flicker light or electric pulses, and the number of pulses (or quantity/frequency of pulses) was integrated over time. Wu et al. (2018) investigated WM of the frequency of visual flicker light and found WM content coded in both, the posterior parietal cortex and the right PFC. Schmidt et al. (2017) found PFC codes of tactile frequency information and Uluç et al. (2018) found similar results when numerosity information was presented as sequentially presented electric pulses. Even though frequency and numerosity are not necessarily the same stimulus property, they are closely related, and appear to rely on similar neuronal processing in the PFC, for which also EEG evidence exists (Spitzer & Blankenburg, 2012; Spitzer, Gloel, Schmidt, & Blankenburg, 2014). In contrast, not finding the PFC in the study at hand but only the IPS, could suggest that the presentation format might play a role for the PFC to exhibit more distinct activation patterns when numerosity is presented in a sequential type of format. A perceptual numerosity study by Cavdaroglu and Knops (2018) directly compared sequential and simultaneous presentation of numerosity. They found numerosity codes in the IPS for simultaneous, but not for sequential presentation and suggested that the differences could be explained by differences in cognitive and WM demands, specifically that the temporal integration of numerosity information might have higher demands than the processing of simultaneous numerosity information. Our data, together with the discussed studies, support the preference of the IPS for simultaneous stimulus presentation, the given null‐finding of the PFC leave open the question if such codes could be detected with high‐resolution imaging.

In sum, our analyses did not find PFC WM codes that would have been detectable with MVPA. However, as this is a null‐finding and not a proof of absence, the role of the PFC for human numerosity WM processing remains a matter for future investigation. Differences between the format of the stimulus material (sequential vs. simultaneous presentation) might contribute to a distinction between the roles of the PFC and the IPS.

## 5 CONCLUSION

This study is the first to dissociate perceptual and WM‐related activity in humans when visual dot arrays are presented as numerosity stimuli. Our results suggest that the right IPS is not only a core region to process numerosity during perception, but also for the retention of numerosity information as WM representations. Finding the strongest effects in the lateral part of the IPS is in line with the suggestion of a functional distinction within the IPS between medial and lateral parts for perceptual and WM subregions. Our study did not find significant effects in the PFC, which might be due to the presentation type of the numerosity information. Previous perceptual and WM studies revealing PFC numerosity codes applied stimuli in which numerosity information had to be integrated over time, such as flicker or vibration frequency. When numerosity information is derived from visual dot stimuli, as in the study at hand, the IPS appears to be the core region to represent numerosity codes for WM.

## CONFLICT OF INTERESTS

The authors declare no competing financial interests.

## Data Availability

The raw data generated during the current study are not publicly available due to data protection laws, but normalized data and the used scripts are available from the corresponding author on request.
